# The relationship between online gaming addiction and the Big-Five personality traits through the mediating role of mental health: The moderating role of gender in university students

**DOI:** 10.1192/j.eurpsy.2026.11916

**Published:** 2026-07-31

**Authors:** M. Sharifi, C. A. Lewis, M. Firoozi, S. Khani, P. Afkari

**Affiliations:** 1 Psychology, Islamic Azad University, Bandargaz Branch, Bandargaz, Iran, Islamic Republic Of; 2 Psychology, Leeds Trinity University, Leeds, United Kingdom; 3 Psychology; 4 Psychology and Educational Sciences, University of Tehran; 5 Psychology, Payam-e-noor University, Tehran, Iran, Islamic Republic Of

## Abstract

**Introduction:**

The role of personality factors in explaining individual difference factors in mental health is well-documented.

**Objectives:**

The present study aimed to investigate the relationship between online gaming addiction and the Big-Five personality traits through the mediating role of mental health, while also examining the moderating role of gender in university students.

**Methods:**

This applied study employed a correlational design using structural equation modeling. The statistical population included all students at the University of Tehran during the 2024–2025 academic year. A total of 306 participants were selected using convenience sampling. The data were collected using the Internet Gaming Disorder Scale, the short form of the Big Five Inventory, and the Cognitive Emotion Regulation Questionnaire. Data analysis was conducted using path analysis in SPSS-24 and LISREL.

**Results:**

The findings revealed significant gender differences in gaming frequency, game types, and gaming platforms. Emotional stability and agreeableness also varied significantly between genders. A significant positive correlation was found between online gaming addiction and the use of maladaptive emotion regulation strategies (p < .001). No significant correlation was observed between online gaming addiction and any of the Big-Five personality dimensions (p > .05). The indirect effects of personality traits on gaming addiction were not significant, and no significant gender differences were found in the paths from emotion regulation strategies to gaming addiction or from personality traits to emotion regulation strategies (p > .05).

**Conclusions:**

These findings may serve as a foundation for developing psychological interventions aimed at preventing and treating online gaming addiction and offer deeper insight into the role of personality traits and emotion regulation in students’ mental health.

**Disclosure of Interest:**

None Declared

